# Antibiotic Resistance Carriage Causes a Lower Survivability Due to Stress Associated with High-Pressure Treatment among Strains from Starter Cultures

**DOI:** 10.3390/ani12111460

**Published:** 2022-06-04

**Authors:** Urszula Zarzecka, Anna Zadernowska, Wioleta Chajęcka-Wierzchowska, Krystyna Wiśniewska, Monika Modzelewska-Kapituła

**Affiliations:** 1 Department of Industrial and Food Microbiology, Faculty of Food Science, University of Warmia and Mazury in Olsztyn Plac Cieszyński 1, 10-719 Olsztyn, Poland; urszula.zarzecka@uwm.edu.pl (U.Z.); anna.zadernowska@uwm.edu.pl (A.Z.); wioleta.chajecka@uwm.edu.pl (W.C.-W.); wikrys@uwm.edu.pl (K.W.); 2 Department of Meat Technology and Chemistry, Faculty of Food Science, University of Warmia and Mazury, Plac Cieszyński 1, 10-719 Olsztyn, Poland

**Keywords:** high-pressure processing, antibiotic resistance, starter cultures, *Lactobacillus*, *Lactococcus*, fitness cost

## Abstract

**Simple Summary:**

High-pressure processing is one of the most promising novel food preservation methods that is increasingly used in the food industry. It is applied in various food products such as dairy, meat, juices, and jams to improve safety and shelf-life by the inactivation of microorganisms and preserving of quality attributes. It is reported that the level of antibiotic resistance may influence the ability of strains to survive stress conditions. In this study, it was investigated if carrying antibiotic resistance genes affects the survival of lactic acid bacteria (*Lactococcus* and the former *Lactobacillus*) strains during high-pressure treatment. It was demonstrated that carrying antibiotic resistance genes strains showed a lower survival in response to pressure than strains carrying up to one resistance gene. The same observations were made for both genera. Appropriately selected parameters of high-pressure treatment may help in the elimination of antibiotic-resistant strains.

**Abstract:**

High-pressure processing is one of the most promising novel food preservation methods that is increasingly used in the food industry. Its biggest advantage is that it is a nonthermal method that ensures the microbiological safety of the product while maintaining other features, including nutritional value. If products made with starter cultures are subjected to high-pressure treatment, the process parameters should be selected so as not to eliminate all microorganisms in the product. The aim of the study was to investigate if carrying antibiotic resistance genes affects the survival of lactic acid bacteria (*Lactococcus* and the former *Lactobacillus*) strains during high-pressure treatment. Survival was assessed using the plate count method. It was shown that the strains carrying antibiotic resistance genes showed a lower survival to high pressure. This might be explained by the phenomenon of fitness cost, consisting in a reduced adaptation of antibiotic-resistant strains related to metabolic expenditure. The obtained results indicate the need for further research in this field and the need to select food processing parameters depending on the strains intentionally included in the food.

## 1. Introduction

High-pressure processing (HPP) is a novel nonthermal food preservation technology gaining increasingly more applications in the food industry. This method is able to improve the food quality, due to its ability to increase food safety and its shelf-life by the inactivation of microorganisms and preserving of quality attributes [1,2]. It involves the application of ultra-high pressure (from 100 MPa to 600 MPa) for 30 s to several minutes [3]. 

HPP treatment affects the functional properties of food proteins. Because of these physicochemical changes, HPP can improve important quality characteristics of food [4,5]. HPP has advantages over thermal methods of food preservation from the sensory and nutritional quality of food point of view. This is due to the use of low temperatures and a short treatment time [6]. Pottier et al. [7] indicated the possibility of using high-pressure processing in a hurdle technology to reduce food additives and salt amounts. They also highlighted the potential of HPP to improve digestibility and to reduce the allergenicity of food. The application of HPP in the food industry has become more popular in recent years. It is applied in various food industries mainly in dairy, meat, juices, and jams production [8].

Starter cultures are the preparation of selected individual or mixed strains. These microorganisms, due to their enzymatic activity, transform substrates into products that give food products certain characteristics [9]. Starter cultures, composed mainly of lactic acid bacteria (LAB), play an essential role in the manufacture of fermented food products. They produce lactic acid, which contributes to food texture, moisture content, flavor, and aroma, and increases food safety [9,10]. Starter cultures are used in the food industry in a variety of food products, including dairy products and fermented meats. Among many LAB, which are used as starter cultures in dairy and meat products, strains of former *Lactobacillus* genus such as *L. sakei*, *L. curvatus*, *L. plantarum*, *L. rhamnosus*, *L. casei*, and *L. acidophilus* might be listed [11,12]. Other than *Lactobacillus* sp., microorganisms are also used in starter cultures, but only in the production of selected foods, for example, coagulase-negative staphylococci such as *Staphylococcus xylosus* and *Staphylococcus carnosus* (meat starter cultures), pediococci such as *Pediococcus pentosaceus* and *Pediococcus acidilactici* (meat starter cultures), and *Propionibacterium* (hard ripened cheese starter cultures) [13].

Available studies on the response of LAB to stress caused by high pressure have focused on changes in the bacterial cell structure, metabolism, growth, and viability. As it was reported previously by Bucka-Kolendo et al. [14], high pressure can induce changes in the bacterial proteome and in protein conformation as well as inhibit their acidifying activity [15]. Previous studies have consistently shown reduced amounts of both *Streptococcus thermophilus* and *Lactobacillus delbrueckii* subsp. *bulgaricus* commonly used as yoghurt starters as a result of HPP. Some authors have also reported the loss of culturability in response to high-pressure treatment; however, it depended on the value of applied pressure. However, preservation of food obtained with the use of starter cultures seems to be a very promising technique. It is reported that lactic acid bacteria are more resistant to high pressure than other microorganisms. This opens up wide opportunities for the development of fermented products with an extended shelf life, as the spoilage pathogens and microorganisms can be effectively eliminated while the microorganisms derived from the starter cultures remain viable [6,16,17,18,19]. 

HHP-induced cell inactivation is mainly related to protein denaturation, which can cause enzyme inactivation and cell protein agglomeration. LAB under stress conditions can activate their defense mechanisms, manifesting in stress-related protein secretion [14].

It is reported that antimicrobial resistance is associated with a fitness cost expressed in growth rate reduction and a lower stress resistance, competitive ability, or virulence potential. This cost is considered as important in the evolutionary dynamics of resistance [20]. One of the factors that could potentially determine the cost of resistance is the genetic basis of resistance. Resistance can result either from chromosomal mutation or the acquisition of mobile genetic elements (MGEs). The fitness cost of resistance differs between MGEs acquisition and mutations. It is reported that resistance resulting from the presence of MGEs has a lower fitness cost than resistance resulting from chromosomal mutations. The low cost of plasmid-associated resistance might help explain why plasmids play a predominant role in the resistance evolution, especially when combined with their ability to horizontally spread [21]. However, the plasmid acquisition fitness cost increases with plasmid resistance range [20]. It has been suggested that in an antibiotic-free environment, the acquisition of antibiotic resistance imposes an overall burden on the resistant organism and it would be conquered by a susceptible wild-type organism [22]. It is presumed that the fitness cost endured by bacteria due to resistance genes is more potent under stressful conditions, which lead to the activation of adaptive responses and significantly reduce the fitness of the strains. Fitness cost generally increases with the stress value and, while imposed by some resistance phenotypes, is more severe under stress than under nonstressful conditions. The biological significance of fitness cost depends not only on the growth rate but also on the colonization capacity and the viability of the strain [23,24].

The aim of the study was to assess the survival of LAB strains used in starter cultures in response to stress related to HPP technology depending on the presence of antibiotic resistance genes. The hypothesis that LAB, used as a component of starter cultures in fermented dairy and meat products, with antibiotic resistance genes differ in survivability during HPP from strains without antibiotic resistance was tested. Due to high costs, HPP is not yet used on a large scale in the food industry, which is why HPP should be treated as a stress factor and this research as basic in nature. However, it is worth paying attention to the technological potential in the future.

## 2. Materials and Methods 

### 2.1. Strains

Strains from the collection of the Department of Food and Industrial Microbiology of the University of Warmia and Mazury in Olsztyn compiled previously were selected for analysis [25]. A total number of 16 strains was selected for analysis. Eight of them carried up to one antibiotic resistance gene, and the remaining eight carried more than one antibiotic resistance gene. Minimum Inhibitory Concentrations (MICs) of antibiotics for each strain were determined using gradient strips on Muller–Hinton agar (Merck, Germany) as described previously [25]. Half of the strains belonged to the *Lactococcus* genus (*L. lactis* ssp. *lactis*: n = 8) and the other half belonged to the former *Lactobacillus* genus (*Latilactobacillus curvatus*: n = 1, *Lacticaseibacillus paracasei*: n = 2, *Lactiplantibacillus paraplantarum*: n = 2, *Lactobacillus delbrueckii*: n = 1, and *Lactobacillus* sp.: n = 2). However, for the sake of uniformity in the content of the article, the authors will use the term *Lactobacillus* to describe the entire group of microorganisms. The characteristics of the strains selected for analysis is summarized in Table 1.

### 2.2. High-Pressure Processing

In order to select the pressure values for the analysis, preliminary studies were carried out at pressures of 300, 400, 500, and 600 MPa. The pressure values were selected based on the analysis of literature data as the most frequently used pressures in food preservation and on the basis of the results of other studies conducted by the research team. Each of these pressure values was applied for 1, 3, and 5 min. After the pressure treatment, a series of ten-fold dilutions were made and the number of colony-forming units (CFU)/mL was determined on de Man, Rogosa, and Sharpe (MRS) agar (Merck, Germany) for the pressurized and unpressurized samples. The analyses were carried out in triplicate on different days. Pressure/time values, which significantly but not completely reduced the number of cells relative to the nonpressurized sample, were selected for the further analysis. 

High-pressure treatment was applied to strains cultures from the stationary growth phase in the amount of 10 mL. The strains were cultured in de Man, Rogosa, and Sharpe (MRS) broth (Merck, Darmstadt, Germany) under anaerobic conditions in 30 °C. The pressurization was carried out in glycol-water solution (1:1, *v*/*v*) using the high-pressure single-chamber U4040 (IWC PAN, Warsaw, Poland, Unipress Equipment Division). Temperature was maintained at 20 ± 3 °C, the pressure rise rate was 300 MPa/min, and the pressure relief time was less than 5 s as described previously [26]. The procedure was subjected to cultures of isolates (10 mL) on MRS broth (Merck, Germany) from the stationary growth phase.

After the stress treatment was completed, the samples were immediately subjected to the next stages of the study.

### 2.3. Survival Analysis

The survival of the strains was checked by the plate count method. For this purpose, a series of ten-fold dilutions were made for each strain before and after exposure to the stress factor, and the number of colony-forming units (CFU)/mL on MRS agar was determined. The number of colonies was counted after 48 h of incubation at 30 °C. The number of CFU/mL was determined immediately after high-pressure treatment and after 72 h of storage at room temperature to determine whether the regeneration of damaged cells occurred.

### 2.4. Statistical Analysis

The results were presented as log CFU/mL mean values with standard deviations (SD). To examine the differences between mean values, analysis of variance was conducted along with Tukey’s test at *p* < 0.05. A three-way ANOVA was applied to evaluate the effect of genus (2 levels: *Lactococcus* and *Lactobacillus*), high-pressure treatment conditions (5 levels: control, 300 MPa/3 min, 300 MPa/5 min, 400 MPa/1 min, 400 MPa/3 min) and number of antibiotic resistance genes (2 levels: up to one and more than one) on HPP survival. Only significant interactions were shown. Statistical analyses were conducted in STATISTICA 13.3 StatSoft^®^ software (Tibco, Inc., Tulsa, OK, USA). 

## 3. Results

After the preliminary studies, the following pressurization parameters were selected for the analysis: 300 MPa/3 min, 300 MPa/5 min, 400 MPa/1 min, and 400 MPa/3 min. The mentioned pressure and time values were selected on the basis of the results obtained in the preliminary study. The selection was made in such a way as to obtain a significant decrease in the number of cells but, simultaneously, to avoid the total inactivation of the bacterial cells. The analysis of the strains survival in response to high-pressure-induced stress showed that strains carrying more than one antibiotic resistance gene showed a lower survival ability. After exposure to 300 MPa, regardless of the pressurization time, the number of CFU/mL decreased by, at most, 1 log cycle, while the application of pressure of 400 MPa led to a reduction in the number of CFU/mL by as much as 8 log cycles. Statistical analysis showed that the decrease in the number of CFU/mL was statistically significant depending on number of antibiotic resistance genes. Looking at the differences in the number of CFU/mL depending on the pressure used and the treatment time, statistically significant differences were only observed after applying a pressure of 400 MPa for 1 and 3 min. No significant differences in survival were observed between *Lactobacillus* and *Lactococcus* strains (Table 2 and Table 3). After storing the samples at room temperature for 72 h, an increase in the number of CFU/mL was observed. The cells number in the samples pressurized at 300 MPa reached the initial level (Table 3 and Table 4).

## 4. Discussion

In this study, a significant reduction in CFU/mL was observed in response to the stress induced by high pressure immediately after the treatment; after 72 h of storage, an increase in the number of CFU/mL was observed; however, the cells number did not reach the initial level when 400 MPa was used. This may suggest that during storage, damaged cells that were unable to grow immediately after pressurization might regenerate. The result was adequate to previous studies [27]. Pega et al. [28] indicated that pressure values below 300–400 MPa applied for up to 10 min were adequate to maintain the required amount of starter LAB in yoghurt. In the study by Li et al. [29], the number of LAB was significantly reduced at 300 MPa for 10 min at 4 °C from 7 to 4–5 log cycles immediately after the treatment, and the number of LAB in kimchi after treatment at 600 MPa was still detected at the level of 3 log cycles. Immediately after pressurization, the number of LAB cells was significantly reduced to 4.6, 3.9, and 2.7 log cycles at 400 MPa for 10, 20, and 30 min, respectively. After storage, an increased LAB count was observed, reaching the initial level after 5 days at 37 °C. Significantly increased counts were also observed during storage at room temperature. Lee et al. [30] also reported a reduction in the LAB counts immediately after pressurization at 400 MPa, but after a 15-day storage at 10 °C, the counts increased and were finally at the initial level. To date, there are many studies about the recovery of bacterial cells injured by high pressure during storage under various conditions. However, it is worth taking into consideration that the increase in LAB counts may result simultaneously from the repair of injured cells and the growth of living bacteria, which survived HPP [31].

An important point often overlooked in the subject of antibiotic resistance is that the development of antibiotic resistance can affect the behavior of microorganisms under food processing conditions. It is suggested that antibiotic-resistant bacteria may show different growth kinetics in laboratory media and may show different resistance to food processing stresses. Nevertheless, there is a lack of research focused on the relationship between antibiotic resistance and food-related stresses, especially in relation to nonpathogenic microorganisms. Therefore, the present study provides insight into this issue. 

The present study also demonstrated that carrying antibiotic resistance genes strains showed a lower survival in response to pressure than strains carrying up to one resistance gene. The same observations were made for both *Lactobacillus* and *Lactococcus* strains. This might be explained by the phenomenon of fitness cost, manifested by a decrease in the stress resistance of strains that are burdened with the presence of antibiotic resistance genes. According to the literature, antibiotic resistance might affect the bacterial physiology including biofilm formation, virulence potential, and survival under stressful conditions [32]. It was found that the genetic basis of resistance plays a key role in determining its costs; plasmid acquisition tends to carry a much smaller cost than evolving resistance de novo by chromosomal mutation [20,33]. Different works support the hypothesis that the plasmid acquisition carrying resistance genes requires novel metabolic resources, which is reflected in a fitness cost [34,35,36]. It is still conceivable that resistant organisms might be outcompeted by susceptible ones [37].

The cost of resistance genes carriage is described widely, while the mechanisms responsible for those costs are still unclear. Several possible explanations have been proposed, mainly metabolic load of the new genes replication and expression. According to this hypothesis, some authors have reported that the deletion of a plasmid, associated with the loss of an antibiotic resistance gene, reduced fitness costs [38,39]. It is reported that the cost of plasmids carrying one resistance gene is lower than the cost of multi-drug resistance plasmids. It suggests that the cost of resistance increases with levels of multidrug resistance. Moreover, a significant correlation between the plasmid cost and the number of antibiotic groups to which that plasmid confers resistance was shown [40,41].

The phenomenon of antibiotic resistance is undesirable not only in relation to pathogenic strains, but also to microorganisms intentionally added to food. As these microorganisms are abundant in food, they risk being involved in the horizontal spread of resistance. The obtained results are optimistic in this respect, as they suggest that appropriately selected parameters of high-pressure treatment may help in the elimination of antibiotic-resistant strains.

The obtained results open the door for further research in this area in order to expand the knowledge in this field, which would allow the demonstration of which genes/mechanisms of resistance have the greatest impact on the survival of strains in response to high-pressure stress.

## 5. Conclusions

The obtained results suggest that the presence of antibiotic resistance genes in the bacterial genome may affect the strain’s ability to survive the effects of stress factors. As a result, survival under the influence of a stress factor becomes an individual feature of the strain. The hypothesis that the presence of antibiotic resistance genes is associated with the ability of LAB strains to survive HPP was proven. It was found that multi-antibiotic-resistant strains were more susceptible to HPP. Further research should be conducted to show whether a similar relationship would be obtained in food due to the fact that it is a much more complex system. Therefore, in the case of food produced with the use of starter cultures, it is important to properly select the parameters of the high-pressure treatment in order to ensure the appropriate number of bacterial cells in the product. 

## Figures and Tables

**Table 1:** Strains used in the study.

Strain	Identification	Antibiotic MICs									Resistance Genes
		AMP	CN	C	TE	E	DA	K	S	VA	
**Carrying more than 1 Resistance Gene**											
LAB-14	*L. lactis* ssp. *lactis*	0.19	0.19	12	48	0.125	1.5	12	0.125	0.19	*tet*K, *aph(3’)-IIIa*, *aac(6′)-Ie-aph(2″)-Ia*, *cat*
LAB-16		0.19	1	8	32	0.125	0.25	8	0.125	0.094	*tet*M, *aph(3’)-IIIa*, *aac(6′)-Ie-aph(2″)-Ia*, *aac(6′)-Ia*
LAB-37		3	0.75	6	24	0.125	0.023	16	0.125	0.19	*tet*M, *aph(3’)-IIIa*, *aac(6′)-Ie-aph(2″)-Ia*
LAB-39		0.25	0.25	8	12	0.19	0.125	6	0.19	0.25	*tet*M, *tet*O, *aph(3’)-IIIa*, *aac(6′)-Ie-aph(2″)-Ia*, *aac(6′)-Ia*
LAB-43	*L. curvatus*	0.125	0.5	16	64	0.125	0.38	6	0.125	-	*tet*M, *tet*O, *aph(3’)-IIIa*, *aac(6′)-Ie-aph(2″)-Ia*, *cat*
LAB-41	*L. paracasei*	1	0.38	32	32	0.125	0.023	4	0.125	-	*tet*W, *aph(3’)-IIIa*, *aac(6′)-Ie-aph(2″)-Ia*, *aac(6′)-Ia*, *cat*
LAB-1	*L. paraplantarum*	0.5	0.75	8	12	0.19	0.5	6	0.19	-	*tet*M, *tet*W, *aph(3’)-IIIa*, *aac(6′)-Ie-aph(2″)-Ia*, *aac(6′)-Ia*, *bla*_OXA_
LAB-11	*L. paraplantarum*	0.25	0.38	32	>256	0.25	0.25	8	0.25	-	*tet*M, *aph(3’)-IIIa*, *aac(6′)-Ie-aph(2′′)-Ia*, *aph(2′)-Ic*, *cat*
**Carrying up to 1 resistance gene**											
LAB-44	*L. lactis* ssp. *lactis*	1.5	1	6	12	0.19	0.032	24	0.19	0.19	-
LAB-59		3	1.5	8	24	0.094	0.023	32	0.094	0.125	-
LAB-68		3	1.5	16	24	0.125	0.023	24	0.125	0.19	-
LAB-15		3	1	8	1.5	0.125	0.047	32	0.125	0.25	-
LAB-42	*L. delbrueckii*	1.5	0.75	6	24	0.25	0.023	16	0.25	-	*msr*A/B
LAB-20	*L. paracasei*	0.5	6	6	1.5	0.064	0.032	48	0.064	-	*cat*
LAB-64	*Lactobacillus* sp.	0.25	0.5	24	32	0.094	0.25	8	0.094	-	*cat*
LAB-71	*Lactobacillus* sp.	0.38	0.75	6	32	0.19	0.094	4	0.19	-	*aph(2′)-Ic*

Abbreviations: MICs—Minimum Inhibitory Concentrations, AMP—ampicillin, CN—gentamicin, C—chloramphenicol, TE—tetracycline, E—erythromycin, DA—clindamycin, K—kanamycin, S—streptomycin, VA—vancomycin.

**Table 2:** Results of the survival analysis of strains in response to high-pressure treatment (mean values of log CFU/mL ± SD).

Strain	log CFU/mL ± SD				
	Control Sample	300 MPa/3 min	300 MPa/ 5 min	400 MPa/1 min	400 MPa/3 min
**Carrying more than 1 resistance gene**					
LAB-14	9.13 ^a^ ± 0.08	8.75 ^a^ ± 0.2	8.23 ^b^ ± 0.07	3.00 ^c^ ± 0.13	1.08 ^d^ ± 0.13
LAB-16	8.91 ^a^ ± 0.08	8.46 ^a^ ± 0.41	8.12 ^b^ ± 0.05	3.78 ^c^ ± 0.14	1.38 ^d^ ± 0.27
LAB-37	8.99 ^a^ ± 0.10	8.48 ^a^ ± 0.05	8.34 ^b^ ± 0.16	4.23 ^c^ ± 0.10	1.26 ^d^ ± 0.07
LAB-39	8.92 ^a^ ± 0.16	8.68 ^a^ ± 0.23	8.13 ^b^ ± 0.13	3.00 ^c^ ± 0.13	1.32 ^d^ ± 0.26
LAB-43	9.33 ^a^ ± 0.15	8.93 ^a^ ± 0.15	8.69 ^b^ ± 0.21	3.08 ^c^ ± 0.07	1.00 ^d^ ± 0.07
LAB-41	8.88 ^a^ ± 0.10	8.48 ^a^ ± 0.08	8.17 ^b^ ± 0.31	3.76 ^c^ ± 0.16	1.30 ^d^ ± 0.33
LAB-1	8.92 ^a^ ± 0.10	8.39 ^a^ ± 0.22	8.24 ^b^ ± 0.23	4.11 ^c^ ± 0.04	1.30 ^d^ ± 0.33
LAB-11	8.91 ^a^ ± 0.11	8.52 ^a^ ± 0.21	8.20 ^b^ ± 0.21	3.00 ^c^ ± 0.20	1.32 ^d^ ± 0.13
**Carrying up to 1 resistance gene**					
LAB-15	8.74 ^a^ ± 0.15	8.56 ^a^ ± 0.29	8.28 ^a^ ± 0.17	5.36 ^b^ ± 0.08	4.18 ^c^ ± 0.17
LAB-44	9.19 ^a^ ± 0.13	9.04 ^a^ ± 0.07	8.81 ^a^ ± 0.20	5.53 ^b^ ± 0.11	4.01 ^c^ ± 0.05
LAB-59	9.32 ^a^ ± 0.09	9.15 ^a^ ± 0.10	8.88 ^a^ ± 0.23	5.25 ^b^ ± 0.05	3.66 ^c^ ± 0.09
LAB-68	8.90 ^a^ ± 0.22	8.72 ^a^ ± 0.13	8.45 ^a^ ± 0.25	5.44 ^b^ ± 0.10	3.51 ^c^ ± 0.05
LAB-42	8.79 ^a^ ± 0.12	8.70 ^a^ ± 0.11	8.62 ^a^ ± 0.09	5.32 ^b^ ± 0.06	4.08 ^c^ ± 0.04
LAB-20	9.31 ^a^ ± 0.11	9.23 ^a^ ± 0.15	9.16 ^a^ ± 0.15	5.45 ^b^ ± 0.09	4.05 ^c^ ± 0.05
LAB-64	9.34 ^a^ ± 0.12	9.24 ^a^ ± 0.09	9.18 ^a^ ± 0.07	5.12 ^b^ ± 0.05	3.62 ^c^ ± 0.11
LAB-71	8.90 ^a^ ± 0.18	8.82 ^a^ ± 0.19	8.74 ^a^ ± 0.23	5.45 ^b^ ± 0.17	3.50 ^c^ ± 0.06

Abbreviations: control sample—before HPP; CFU—colony-forming units, SD—standard deviation; ^a–d^ mean values in rows with different letters differ significantly at *p* < 0.05.

**Table 3:** Effect of the genus, pressure conditions, and number of antibiotic resistance genes on survival of the starter culture components (mean values of log CFU/mL ± SD).

Time (h)	Genus		HPP Conditions				No of ARGs		*p*-Value			
	*Lactococcus*	*Lactobacillus*	300 MPa /3 min	300 MPa /5 min	400 MPa /1 min	400 MPa /3 min	≤1	>1	G	HPP	No of ARGs	HPP*ARGs
0	6.62 ± 2.75	6.67 ± 2.81	8.75 ^a^ ± 0.28	8.51 ^a^ ± 0.35	4.42 ^b^ ± 1.01	2.5 ^c^ ± 1.31	7.18 ± 2.20	6.11 ± 3.17	NS	***	***	***
72	6.33 ± 2.68	6.36 ± 2.73	8.89 ^a^ ± 0.26	8.69 ^a^ ± 0.29	4.87 ^b^ ± 1.04	2.92 ^c^ ± 1.42	7.02 ± 2.05	5.67 ± 3.09	NS	***	***	***

Abbreviations: HPP—high-pressure processing, ARGs—antibiotic resistance genes, G—genus, ^a–c^ mean values in rows with different superscripts differ significantly at *p* < 0.05 within HPP group; NS—no significant differences (*p* > 0.5); *** significant differences at *p* < 0.001.

**Table 4:** Results of the survival analysis of strains in response to high-pressure treatment after 72 h of storage at room temperature (mean values of log CFU/mL ± SD).

Strain	log CFU/mL ± SD				
	Control Sample	300 MPa/3 min	300 MPa/5 min	400 MPa/1 min	400 MPa/3 min
**Carrying more than 1 resistance gene**					
LAB-14	9.13 ^a^ ± 0.08	8.85 ^a^ ± 0.11	8.52 ^a^ ± 0.11	3.48 ^b^ ± 0.04	1.40 ^c^ ± 0.12
LAB-16	8.91 ^a^ ± 0.08	8.60 ^a^ ± 0.09	8.45 ^a^ ± 0.06	4.01 ^b^ ± 0.01	1.61 ^c^ ± 0.09
LAB-37	8.99 ^a^ ± 0.10	8.74 ^a^ ± 0.09	8.52 ^a^ ± 0.03	4.71 ^b^ ± 0.03	1.51 ^c^ ± 0.16
LAB-39	8.92 ^a^ ± 0.16	8.80 ^a^ ± 0.06	8.44 ^a^ ± 0.15	3.46 ^b^ ± 0.07	1.70 ^c^ ± 0.08
LAB-43	9.33 ^a^ ± 0.15	8.95 ^a^ ± 0.05	8.72 ^a^ ± 0.21	3.49 ^b^ ± 0.04	1.40 ^c^ ± 0.18
LAB-41	8.88 ^a^ ± 0.10	8.61 ^a^ ± 0.07	8.42 ^a^ ± 0.07	4.12 ^b^ ± 0.08	1.60 ^c^ ± 0.08
LAB-1	8.92 ^a^ ± 0.10	8.65 ^a^ ± 0.11	8.49 ^a^ ± 0.05	4.70 ^b^ ± 0.05	1.56 ^c^ ± 0.10
LAB-11	8.91 ^a^ ± 0.11	8.76 ^a^ ± 0.13	8.33 ^a^ ± 0.07	3.30 ^b^ ± 0.08	1.60 ^c^ ± 0.07
**Carrying up to 1 resistance gene**					
LAB-15	8.74 ^a^ ± 0.15	8.07 ^a^ ± 0.05	8.63 ^a^ ± 0.09	5.89 ^b^ ± 0.15	4.71 ^c^ ± 0.06
LAB-44	9.19 ^a^ ± 0.13	9.11 ^a^ ± 0.07	8.97 ^a^ ± 0.09	5.91 ^b^ ± 0.05	4.67 ^c^ ± 0.13
LAB-59	9.32 ^a^ ± 0.09	9.24 ^a^ ± 0.09	8.97 ^a^ ± 0.13	5.75 ^b^ ± 0.12	3.93 ^c^ ± 0.04
LAB-68	8.90 ^a^ ± 0.22	8.85 ^a^ ± 0.07	8.73 ^a^ ± 0.11	5.90 ^b^ ± 0.06	3.95 ^c^ ± 0.12
LAB-42	8.79 ^a^ ± 0.12	8.72 ^a^ ± 0.05	8.64 ^a^ ± 0.11	5.73 ^b^ ± 0.07	4.71 ^c^ ± 0.03
LAB-20	9.31 ^a^ ± 0.11	9.29 ^a^ ± 0.15	8.98 ^a^ ± 0.09	5.92 ^b^ ± 0.13	4.70 ^c^ ± 0.06
LAB-64	9.34 ^a^ ± 0.12	9.26 ^a^ ± 0.13	8.99 ^a^ ± 0.21	5.71 ^b^ ± 0.07	3.83 ^c^ ± 0.23
LAB-71	8.90 ^a^ ± 0.18	8.86 ^a^ ± 0.07	8.78 ^a^ ± 0.06	5.93 ^b^ ± 0.02	3.94 ^c^ ± 0.13

Abbreviations: control sample—before HPP; CFU—colony-forming units, SD—standard deviation; ^a–c^ mean values in rows with different letters differ significantly at *p* < 0.05.

## Data Availability

The dataset used during this study is available from the author upon reasonable request.
